# Epidermal loss of Gα_q_ confers a migratory and differentiation defect in keratinocytes

**DOI:** 10.1371/journal.pone.0173692

**Published:** 2017-03-16

**Authors:** Colleen L. Doçi, Constantinos M. Mikelis, Juan Luis Callejas-Valera, Karina K. Hansen, Alfredo A. Molinolo, Asuka Inoue, Stefan Offermanns, J. Silvio Gutkind

**Affiliations:** 1 College of Arts and Sciences, Marian University Indianapolis, Indianapolis, Indiana, United States of America; 2 Department of Biomedical Sciences, School of Pharmacy, Texas Tech University Health Sciences Center, Amarillo, Texas, United States of America; 3 Department of Pharmacology and Moores Cancer Center, University of California San Diego, La Jolla, California, United States of America; 4 Oral and Pharyngeal Cancer Branch, National Institute of Dental and Craniofacial Research, National Institutes of Health, Bethesda, Maryland, United States of America; 5 Department of Pathology, University of California San Diego, La Jolla, California, United States of America; 6 Graduate School of Pharmaceutical Sciences, Tohoku University, Sendai, Miyagi, Japan; 7 PRESTO, Japan Science and Technology Agency (JST), Kawaguchi, Saitama, Japan; 8 Department of Pharmacology, Max-Planck Institute for Heart and Lung Research, Bad Nauheim, Germany; Augusta University Medical College of Georgia, UNITED STATES

## Abstract

G-protein coupled receptors (GPCRs), which activate heterotrimeric G proteins, are an essential class of transmembrane receptors that are responsible for a myriad of signaling events in normal and pathologic conditions. Two members of the G protein family, Gα_q_ and Gα_11_, activate one of the main GPCR pathways and function as oncogenes by integrating mitogen-stimulated signaling cascades that are active under malignant conditions. Recently, it has been shown that targeted deletion of Gα_11_ and Gα_q_ from endothelial cells impairs the Rho-mediated formation of focal adherens junctions, suggesting that Gα_11/q_ signaling may also play a significant role in cytoskeletal-mediated cellular responses in epithelial cells. Indeed, combined deletion of Gα_11_ and Gα_q_ confers a significant migratory defect in keratinocytes that delays cutaneous wound healing in an *in vivo* setting. This delay can be attributed to a defect during the reepithelialization phase due to significantly attenuated migratory capacity of Gα_q_-null keratinocytes under combined Gα_11_ deficiency. In fact, cells lacking Gα_11/q_ demonstrate a severely reduced ability to respond to mitogenic and migratory signals in the microenvironment, leading to inappropriate and premature terminal differentiation. These results suggest that Gα_11/q_ signaling pathways may be critical for integrating mitogenic signals and cytoskeletal function to achieve normal physiological responses. Emergence of a malignant phenotype may therefore arise from both under- and overexpression of Gα_11/q_ signaling, implicating its upstream regulation as a potential therapeutic target in a host of pathologic conditions.

## Introduction

The signal transduction mechanisms in the cell that mediate gene expression, cell survival, and growth rely heavily on the function of the largest family of cell-surface molecules, the G-protein coupled receptors (GPCRs) [1]. The GPCR family transduces signals from the cellular microenvironment via receptor coupling and subsequently activating heterotrimeric G proteins. This activation in turn leads to the second-messenger systems that influence transcription, translation, viability, and normal and malignant cell growth. Within the heterotrimeric G proteins, activating mutations in multiple members of the Gα subunit family have been shown to function as oncogenes. Among the heterotrimeric G proteins, Gα_q_ and Gα_11_ share around 90% amino acid homology and are the main components of the Gq family of alpha proteins [2]. Gain of function in Gα_q_ and Gα_11_ leads oncogenic transformation [3, 4] and is particularly prevalent in the development and progression of uveal melanoma, where Gα_q_ and Gα_11_ share activating mutations and are currently being evaluated for both diagnostic and therapeutic purposes [5–8]. However, relatively little is known about the pathological consequences of Gα_11_ and Gα_q_ loss.

*GNAQ* (Gα_q_) and *GNA11* (Gα_11_) comprise the Gq family of alpha proteins that transduces many of the mitogen-stimulated GPCR signaling cascades. Gα_q_ stimulates Phospholipase C (PLC) to achieve downstream activation of Protein Kinase C (PKC) and extracellular signal-related kinase (ERK) [9]. Combined deletion of Gα_11_ and Gα_q_ leads to embryonic lethality at day 11 due to myocardial hypertrophy, while global deletion of *GNA11* alone causes no apparent behavioral and morphological defects and global deletion of *GNAQ* alone leads to ataxia and impaired motor control [10, 11]. Thus, due to their compensatory functions, investigation into the role of *Gnaq* and *Gna11* requires conditional deletion of one of these genes in the context of total loss of its compensatory family member. Using this approach, it has been shown that conditional deletion of Gα_q_ in the context of *Gna11* abolishes the embryonic lethality and hypertrophic response in cardiomyocytes observed previously [12]. This approach has been also used to demonstrate that loss of Gα_q_ from endothelial cells under combined Gα_11_ deficiency impairs the Rho-mediated formation of focal adherens junctions after histamine stimulation [13]. Gα_11_ and Gα_q_ have also been found to play a large role in the genetics of the skin [14, 15]. However, it is unclear if cutaneous targeted deletion of Gα_11_ and Gα_q_ may also have an impact on epidermal cytoskeletal integrity and function.

Here, we show that targeted deletion of Gα_11_/Gα_q_ in the skin does not result in cancer formation, but rather underlies an acute migratory defect in keratinocytes. While not deleterious under physiologic conditions, this defect manifests as a severe attenuation in the rate of cutaneous wound healing. We further demonstrate that this migratory defect may be partly attributed to an inability of Gα_11/q_-null keratinocytes to respond to the mitogenic signals that mediate the transition between a differentiated, static cell and a more stem-like phenotype capable of engaging in highly migratory reepithelialization programs. These results suggest that the Gα_11/q_ signaling pathway may be an important mediator of pathologic stress, where low or inactivated Gα_q_ results in a chronic wound phenotype that may contribute to malignant transformation. Therefore, understanding the factors that contribute to Gα_q_ expression and regulate its function may have a broad impact in a variety of pathologic disease conditions and open new therapeutic avenues in the future.

## Materials and methods

### Mice

All animal studies were carried out according to NIH-approved protocols, in compliance with the Guide for the Care and Use of Laboratory Animals. All mice used were in C57BL/6 background, 8 weeks old or older and both males and females were used for experiments. The generation of null alleles of the genes encoding Gα_11_ (*Gna11*) and of the floxed alleles of the genes encoding Gα_q_ (*Gnaq*) have been described previously [16]. For the experiments probing Gα_11_ deficiency only, *Gna11*-/- mice and littermate controls were used. Pups were obtained from a heterozygote mating where *Gna11*^+/-^ x *Gna11*^+/-^ animals were crossed, and upon genotyping the *Gna11*^+/+^ and *Gna11*^-/-^ littermates were selected for experiments. Epidermal-specific Gα_q_ knockouts were obtained by crossing the aforementioned mice with mice carrying a constitutive Cre-mediated recombination system driven by the K14 promoter (K14-Cre, [17]), where *Gna11*^-/-^*Gnaq*^f/f^ K14-Cre^+/-^ mice were crossed with *Gna11*^-/-^*Gnaq*^f/f^ K14-Cre^-/-^ such that all pups were deficient for Gα_11_ and double floxed for Gα_q_. Expression of Ga_q_ was therefore driven by inheritance of the K14-Cre promoter, where 50% of the pups expressed the K14-Cre promoter (*Gna11*^-/-^*Gnaq*^f/f^ K14-Cre^+/-^, referred to as Gα_11_KO/Gα_q_-eKO) and were the experimental group, while the other 50% did not expressing the K14 promoter (*Gna11*^-/-^*Gnaq*^f/f^ K14-Cre^-/-^, referred to as Gα_11_KO/Gα_q_-WT) and were used as controls. Both males and female mice were used for the experiments.

### Reagents and antibodies

Cell culture surfaces were coated with 10 μg/ml of poly-L-lysine (Sigma), type I rat tail collagen (BD Biosciences) or fibronectin (Invitrogen). Recombinant human TGF-β1 from Peprotech. Deep space black and warp red chromogen kits were from Biocare medical. Antibodies for western blotting and immunohistochemistry were used as described in Table 1.

**Table 1:** Antibodies and experimental conditions. This table details the antibodies, their formulation, experimental application, dilution, and source for all experiments in this manuscript.

Antibody	Conjugation	Primary/Secondary	Application	Dilution	Company
Actin	None	Primary	Western	1:5000	Cell Signaling Technology
Goat a-rabbit	Biotinylated	Secondary	IHC	1:400	Vector Laboratories
Goat a-rabbit	HRP	Secondary	Western	1:40,000	Southern Biotech
Goat a-rat	Biotinylated	Secondary	IHC	1:400	Vector Laboratories
Gα_q_	None	Primary	Western	1:1000	Santa Cruz Biotechnology
K10	None	Primary	IHC	1:2000	Covance
K13	None	Primary	IHC	1:5000	Abcam
K14	None	Primary	IHC	1:1000	Covance
Ki67	None	Primary	IHC	1:50	Dako
Phospho-SMAD2	None	Primary	Western	1:1000	Cell Signaling Technology
SMAD2	None	Primary	Western	1:1000	Cell Signaling Technology

### Cell lines, keratinocyte cell culture, and transfections

All cells were cultured at 37°C in the presence of 5% CO_2_. HEK293ΔGα_q_ cells, which lack functional Gα_q_ and Gα_11_, were described previously [18]. Cells were cultured in DMEM (Invitrogen) containing 10% fetal bovine serum (FBS; Sigma-Aldrich) and antibiotic/antimycotic solution (Sigma-Aldrich). Cells were transfected with Turbofect (Fermentas) according to the manufacturer’s instructions. Fibroblasts and keratinocytes from newborn mouse skin were isolated as previously described [19]. Keratinocytes were cultured in defined keratinocyte serum-free medium (KSFM; Invitrogen) and subdermal fibroblasts were cultured in DMEM 10% FBS. Cells were transfected with 10ng/cm^2^ of either pCAGGS-Gα_q_ or vector control and/or 200ng/cm^2^ LifeAct-GFP and assessed 48 hours post transfection.

### Immunoblot analysis

Cells were lysed in RIPA buffer [50 mM Tris-HCl, 150 mM NaCl, 1% NP-40, 0.5% sodium deoxycholate] supplemented with protease and phosphatase inhibitors (Thermo Fisher) for 15 minutes on ice, scraped, and then sonicated for 3x10s on 30% power. Lysates were cleared by centrifugation at 10,000*xg* for 15 minutes at 4°C and concentration was determined using Bio-Rad DC protein assay. Ten micrograms total protein was separated by SDS-PAGE and transferred to PVDF membrane overnight at 4°C. Membranes were blocked for 1 hour at room temperature in 5% milk in TBST and then probed with primary antibodies overnight at 4°C. Membranes were washed four times in TBST, probed with HRP-conjugated secondary antibodies for 1h at RT in 5% milk, washed four times in TBST, and detected using chemiluminescent substrate (Millipore) with the FluorChem E system image analyzer (Cell Biosciences, Santa Clara, CA)

### Histopathologic and immunohistologic analysis

Formalin-fixed paraffin-embedded (FFPE) tissue slides were deparaffinized in 100% xylene, hydrated through a series of graded alcohols (100%, 95%, 80%, and 70%), and incubated in sodium citrate buffer (pH6.0) to unmask the antigen. Endogenous peroxidase activity was blocked by incubate with 3% H_2_O_2_, blocked in PBS with 3% BSA, and then incubated with primary antibody in blocking buffer overnight at 4°C. The slides were washed in PBS three times, incubated with a biotin-conjugated secondary antibody at room temperature for 30 minutes followed by the avidin-biotin complex (Vector Stain Elite Standard, ABC kit, Vector Laboratories) for 30 minutes at room temperature. For single stained slides, the slides were washed and developed in 3,3′-diaminobenzidine (Sigma FASTDAB tablet, Sigma Chemical) under microscopic observation. The reaction was stopped in tap water and the tissues were counterstained with hematoxylin, dehydrated, and mounted. For double stained images, slides were developed with Deep Space Black chromogen kit according to manufacturer’s instructions (Biocare Medical). The reaction was stopped in tap water, washed with TBS, blocked in TBS with 3% BSA, and then incubated with primary antibody in blocking buffer overnight at 4°C. The slides were washed in TBS three times, incubated with alkaline phosphatase-conjugated secondary antibody at room temperature for 30 minutes followed by the AP complex (Vector Stain Elite AP, ABC kit, Vector Laboratories) for 30 minutes at room temperature. Slides were washed and developed using Warp Red chromogen kit according to manufacturer’s instructions (Biocare Medical) under microscopic observation. Images were taken using Scanscope (Aperio). Quantification of slides stained for Ki67 was performed using Aperio Scanscope software and the Nuclear v9 algorithm, while quantification of K10 and K13 was performed using IHC Membrane v9 algorithm. H-scores were determined as the product of the staining intensity (0, absent; 1, weak staining; 2, moderate staining; and 3, strong staining) multiplied by the percentage of positive cells quantified.

### Proliferation assay (EdU incorporation assay)

To visualize individual cells synthesizing DNA we used the Click–It kit (Invitrogen) according to manufacturer’s instructions. This kit allows for robust statistical analysis in small populations of cells while utilizing the highly-specific labeling methodology of BrdU or [^3^H]-thymidine incorporation. Briefly, subconfluent cells were grown in 96-well plates in quadruplicate, starved, and transferred to complete media for 16 h. Then EdU (10 μM) was incorporated for 4 h and cells were fixed in a 4% paraformaldehide/PBS solution (Electron Microscopy Sciences, PA USA). Samples were counterstained with Hoescht 33342 and visualized under Axio Imager Z1 microscope equipped with ApoTome system controlled by AxioVision software (Carl Zeiss).

### Colony formation assay

For the colony formation analysis, cells were plated in 6-well plates at 100 live cells/well (counted with trypan blue) and cultured until visible colonies around 1–2 mm in diameter appeared in control wells. The cells were then stained with crystal violet fixing solution (0.05% crystal violet in PBS with 4% paraformaldehyde) for 30 min RT, washed with water, and the resulting colonies were quantified by size and number per plate with ImageJ.

### Scratch wound closure

Scratch assays were performed with keratinocytes or fibroblasts grown to confluence on fibronectin-coated wells and starved overnight. Scratches were made with a plastic pipette tip across the diameter of each well. Phase contrast images of the wound were obtained every 6 h for fibroblasts and every 24 h for the keratinocytes, Quantitative analysis of uncovered wound area was performed using the Axiovision Rel. 4.7 (Carl Zeiss, Thornwood, NY). Calculations were based on three replicates per experiment from three independent experiments.

### Data mining and GEO analysis

Data regarding the expression levels of migratory genes was downloaded from the Gene Expression Omnibus (http://www.ncbi.nlm.nih.gov/geo/, accessed June 30, 2015) for GSE8531. Significant genes, defined as those with a p-value less than 0.05 and logFC value >2 or <-2, were inputted into the ENRICHR algorithm (http://amp.pharm.mssm.edu/Enrichr/, accessed June 30, 2015). The GO Molecular Function ontology was downloaded and represented according to the internally calculated p-value.

### Migration assay

Cells were serum starved for six hours and placed in the top well of a Boyden chamber while serum-free media or serum-free media supplemented with 5% FBS, 1 ng/ml TGF-β1, or 10 ng/ml TGF-β1 was placed in the bottom well. The two chambers were separated by an 8 μm pore polyvinylpropylene-free membrane coated with 10 μg/ml collagen. Cells were incubated at 37°C in a humidified chamber overnight. The next day, the apparatus was disassembled and the membrane was fixed for 30 minutes in methanol at room temperature. The membrane was then stained with hematoxylin for 20 minutes at RT, washed three times with distilled water, mounted face down on glass slides, and nonmigrated cells were scraped off with cotton swabs. Membranes were scanned and quantified using ImageJ. Calculations were based on 6 imaging fields each from three independent experiments. Statistical significance was determined using Student’s t-test.

### Immunofluorescence

Cells were transfected with LifeAct-GFP and either empty vector or Gα_q_ and grown on cover slips coated with collagen I. Cells were starved for 6 hours and then treated for 3 hours with either 0, 1, or 10 ng/ml TGF-β1. Cells were fixed with 4% paraformaldehyde, washed in PBS, and mounted on uncharged slides. The images were taken using an Axio Imager Z1 microscope equipped with an ApoTome system.

### Wound healing assay and experimental mice

This study was approved by the Animal Care and User Committee, according to National Institutes of Health (NIH) animal study protocols approved by the Animal Care and Use Committee protocol 12–667, National Institute of Dental and Craniofacial Research, in compliance with the “Guide for the Care and Use of Laboratory Animals”. Animals were housed on 12-h light/dark cycles and received food, standard rodent chow, and water ad libitum in compliance with Association for Assessment and Accreditation of Laboratory Animal Care International guidelines. The animals were observed daily by the investigators and animal care staff. Any animals displaying signs of discomfort, wasting, ruffled hair coat, hunching, or other signs indicative of distress were treated appropriately to alleviate discomfort or euthanized if recommended by animal care staff or the facility veterinary. Mice were anesthetized and dorsal surfaces were shaved and cleaned with betadine topical solution. Fifteen millimeters full-thickness incisional skin wounds were made in the mid-dorsal area. The wound sites were monitored and measured daily utilizing wound closure as endpoint as previously [20]. At indicated time points, the wound field was excised and fixed in aqueous buffered zinc formalin for 24 h, transferred to 70% ethanol, paraffin embedded and sectioned. Wound size was determined as the factor of wound width and length, normalized to the wound area at Day 0. Wound half-life was determined from the nonlinear regression of wound closure over time per animal.

### Statistical analysis

All analyses were performed with a minimum of three technical replicates per experiment from three independent experiments and the means obtained were used for ANOVA or independent t-tests. Additional technical and biological replicates were used as indicated. Statistical analyses, variation estimation and validation of test assumptions were carried out using the Prism 6 statistical analysis program (GraphPad). Asterisks denote statistical significance (nonsignificant or NS, P > 0.05; *P < 0.05; **P < 0.01; and ***P < 0.001). All data are reported as mean ± standard error of the mean (s.e.m.).

## Results

### Epidermal integrity is maintained under homeostasis despite targeted deletion of Gα proteins

**Fig 1:**
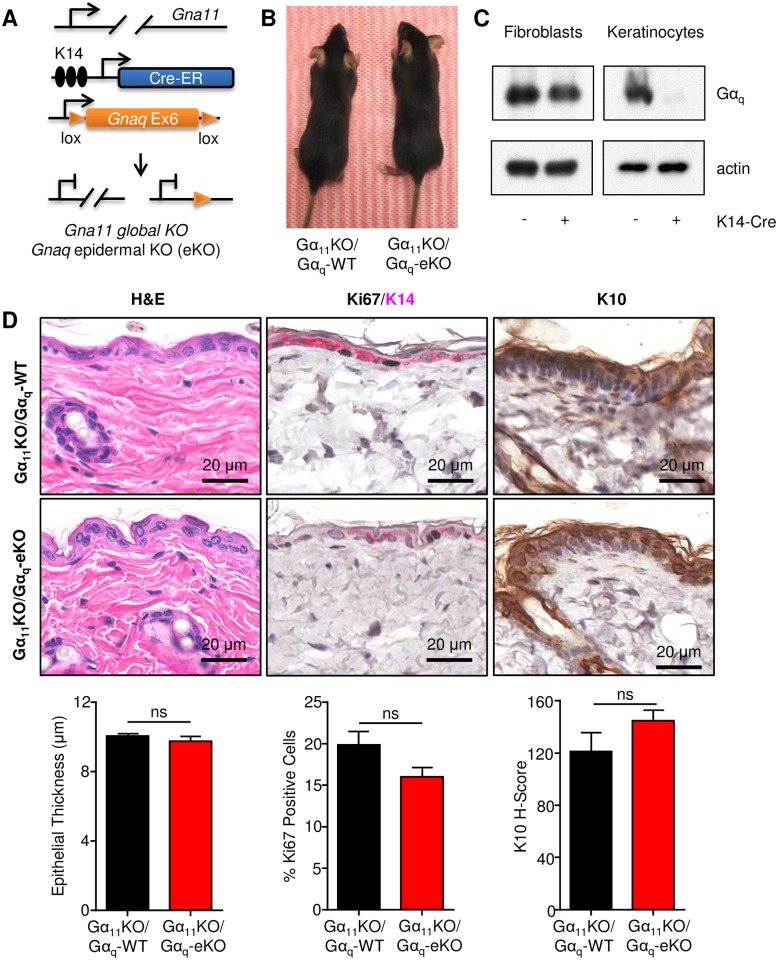
Mice with epidermal loss of Gα_q_ do not demonstrate phenotypic defects under normal conditions. A) Schematic representation of the animal model used to derive a delete *Gnaq* exon 6 (Ex6) from the basal epidermal compartment concomitant with global deletion of *Gna11*. B) Representative pictures of Gα_11_KO/Gα_q_-WT control and Gα_11_KO/Gα_q_-eKO mice. C) Western blot demonstrating Gα_q_ deletion in the epidermal keratinocytes but not fibroblasts after expression of K14-Cre. D) Normal skin samples from Gα_11_KO/Gα_q_-WT control and Gα_11_KO/Gα_q_-eKO animals (N = 4 each) were examined for histology (H&E), proliferation (Ki67), and differentiation (K10).

We have recently shown that activating mutations in Gα_q_ function as drivers of oncogenesis in uveal melanoma and are also found in a variety of other malignancies [21, 22], while deletion of *Gnaq* in the context of *Gna11* deficiency appears to have significant defects in developmental and Rho-mediated endothelial cell functions [11, 13]. Deletion of *Gnaq* alone results in ataxia, impaired motor control, and skin pigmentation [10, 11, 14, 23]. However, a specific functional role for Gα_q_ proteins in the skin has not been identified partially due to the compensatory roles that Gα_q_ and Gα_11_ play *in vivo*. To determine if Gα_q_ proteins might have a specific effect on epidermal function, we used a mouse model harboring global deletion of Gα_11_ (Gα_11_KO) and targeted deletion of Gα_q_ in the skin (Gα_q_-eKO) driven by the K14 promoter (Fig 1A). Gα_11_KO/Gα_q_-eKO animals displayed no gross abnormalities (Fig 1B) despite complete deletion of Gα_q_ proteins from the skin (Fig 1C). Histologically, both littermate controls bearing global Gα_11_ deletion but expressing WT Gα_q_ and Gα_11_KO/Gα_q_-eKO animals demonstrated no differences in epidermal thickness (Fig 1D). There was a slight decrease in Ki67 proliferating cells that corresponded with a slight increase in K10^+^ differentiated cells in Gα_11_KO/Gα_q_-eKO animals, suggesting that there might be a stronger tendency towards terminal differentiation within these cells (Fig 1D). However, these animals were largely phenotypically normal under homeostatic conditions and did not present the endogenous tumorigenic susceptibility of the Gα_s_ model.

### Loss of Gα_q_ confers a strong migratory defect in keratinocytes

**Fig 2:**
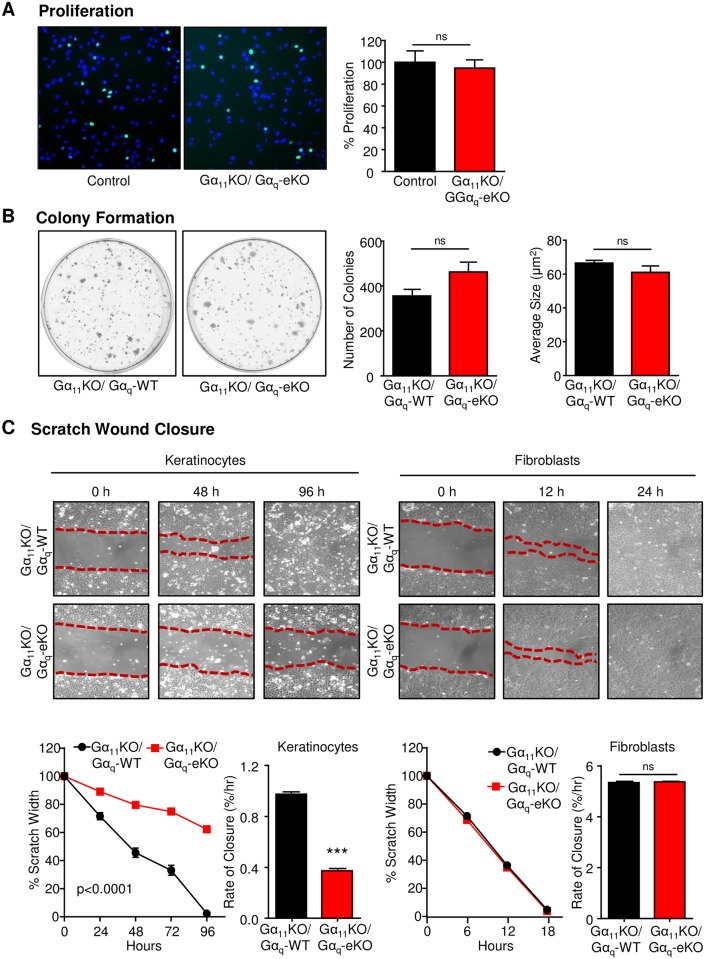
Gα_11/q_-null keratinocytes have normal proliferative and stem-like phenotypes but demonstrate a significant migratory defect. Keratinocytes from Gα_11_KO/Gα_q_-WT control and Gα_11_KO/Gα_q_-eKO animals were assayed for proliferation (A), colony formation (B) and scratch wound closure (C). To confirm the migratory defect, keratinocyte and fibroblast wound closure from Gα_11_KO/Gα_q_-WT control and Gα_11_KO/Gα_q_-eKO were quantified. Statistical significance was determined using Student’s t-test, *p<0.05, **p<0.01, ***p<0.001.

To determine if any defect might be present within Gα_11/q_-null keratinocytes, we isolated cells from mice and analyzed them according to the key hallmarks of carcinomas, namely increased proliferation, increased colony formation, and increased migration. Similar to what we observed in histologic sections, Gα_11/q_-null keratinocytes did not demonstrate any differences in proliferation (Fig 2A). Similarly, no statistical differences could be detected in the size or number of colonies (Fig 2B). However, a remarkable phenotype was observed upon induction of migration, where Gα_11/q_-null keratinocytes had a profound defect in their ability to migrate after scratch wounding (Fig 2C). Over a 96-hour time course, control keratinocytes migrated to close the gap formed after a scratch wound assay, while Gα_11/q_-null keratinocytes were able to close less than half of the gap in the same time frame (Fig 2C). This was due specifically to loss of Gα_11/q_ and not due to loss of Gα_11_ alone, as Gα_11_-null fibroblasts isolated from the same mice showed no such defect and were identical to fibroblasts from control mice (Fig 2C). Together, this suggested that rather than promoting a carcinogenic phenotype, deletion of Gα_q_ from the epidermis contributed to a migratory defect. To determine if a similar phenotype had been observed in other studies, we searched for gene expression data related to alteration of a migration phenotype in keratinocytes. By annotating the GEO dataset from a study that used TGF ligands to assess migration-specific genes [24], we found that among the ten most statistically significantly altered ontologies amongst regulated genes, nine were related to Gα protein signaling events and specifically to events associated with Gα_q_ activity, including phospholipase C activity (S1 Fig).

**Fig 3:**
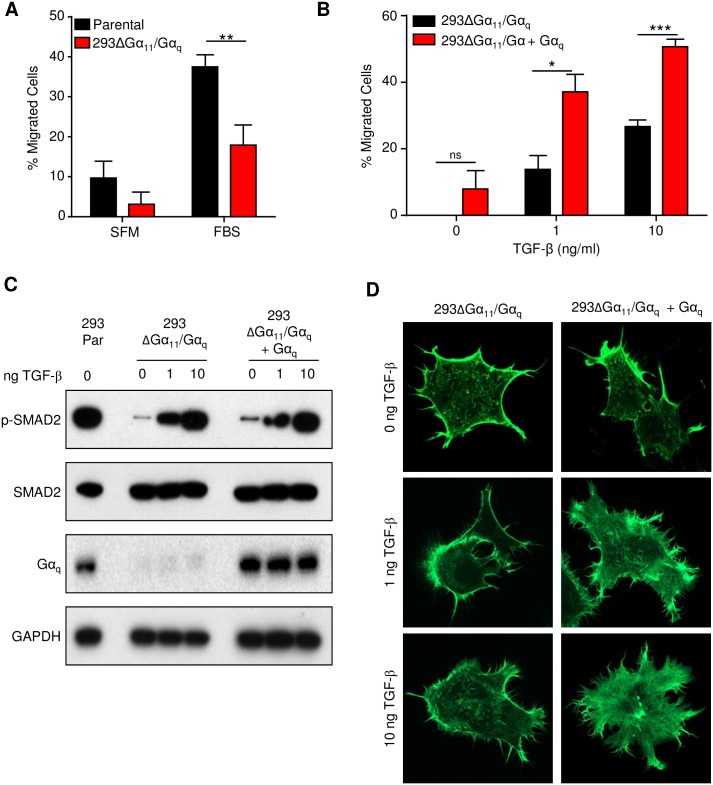
Gα_q_ enhances TGF-β stimulated migration and cytoskeletal remodeling. A) Parental and Gα_11/q_-null HEK293 cells were assessed for migration towards serum free media (SFM) or 5% fetal bovine serum (FBS). B) Gα_11/q_-null or Gα_11/q_-null with transfected, reconstituted expression of Gα_q_ were allowed to migrate towards 0, 1, or 10 ng/ml TGF-β1. C) Western blot demonstrating efficacy of TGF-β signaling in Gα_11/q_-null and reconstituted cells after 30 minutes treatment with 0, 1, or 10 ng/ml TGF-β1. D) Gα_11/q_-null and reconstituted cells co-expressing LifeActGFP were treated with 0, 1, or 10 ng/ml TGF-β1 for 3 hours and assessed for cytoskeletal changes. Statistical significance was determined by Student’s t-test, *p<0.05, **p<0.01, ***p<0.001.

TGF-β has been largely characterized in cell migration due to its pleiotropic effects in a wide variety of cell types and in particular its contribution to proliferation and migration in cutaneous injury [25–27]. Taking advantage of the CRISPR targeted deletion system, we tested the possibility that loss of Gα_q_ might be necessary for integrating migration stimulatory signals. First, we compared parental HEK293 cells with Gα_11_/Gα_q_ CRISPR-deleted HEK293 migration and found that consistent with our previous observations, the Gα_11/q_-null cells migrated significantly less under serum-starved and FBS-stimulated conditions (Fig 3A). To further investigate whether this was a migration-specific defect due to Gα_q_ expression and not due to competing mitogen signaling or clonality issues from the CRISPR cell cloning, we reintroduced Gα_q_ with low levels of transfection plasmid or used empty vector as a control and then assessed their TGF-β-dependent migration. Gα_11/q_-null cells showed a modest, dose-dependent response to TGF-β, indicating that they are capable of responding to TGF-β signaling (Fig 3B). However, reintroduction of Gα_q_ resulted in a far more robust response to TGF-β and these cells migrated significantly more than empty vector controls (Fig 3B). To confirm that this was not due to a defect in TGF-β signaling, we stimulated the cells for 30 minutes and then performed western blots for downstream targets of TGF-β, namely SMAD2. Both the Gα_q_-expressing and vector control cells responded to TGF-β signaling to approximately the same degree, as indicated by accumulation of p-SMAD2 (Fig 3C). This suggests that TGF-β does not signal directly through Gα_q_, but rather that Gα_q_ participates at some level in orchestrating the signaling cascade that is initiated upon TGF-β in order to facilitate migration. Migration requires reorganization of the actin cytoskeleton, so we imaged vector control and Gα_q_-restored cells after treatment with TGF-β via fluorescently-labelled actin. Here, vector control cells acquired a ruffled cytoskeletal appearance at high concentrations of TGF-β, but Gα_q_-restored cells were able to achieve a more efficient reorganization of their cytoskeleton at low doses of TGF-β (Fig 3D). Together, these results demonstrate that loss of Gα_q_ confers a strong migratory defect in epithelial cells that is specific to migration-related signaling events and independent of other growth related signaling cues.

### Gα_q_-induced migratory defects in keratinocytes leads to significant delay in cutaneous wound healing

**Fig 4:**
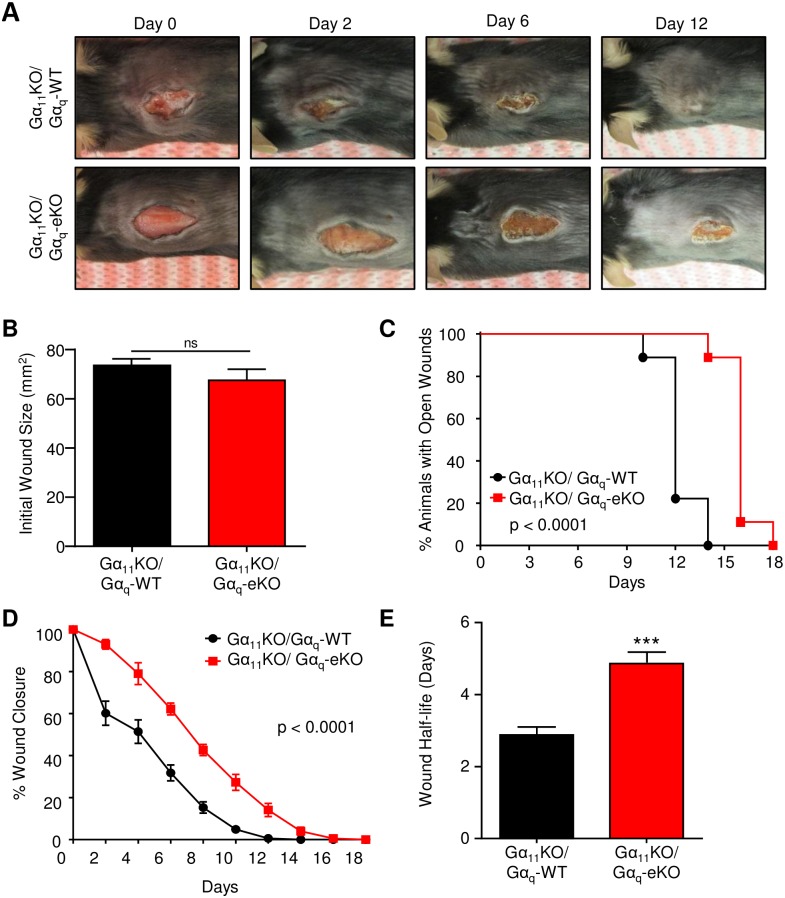
Gα_11_KO/Gα_q_-eKO mice have delayed wound healing. Gα_11_KO/Gα_q_-WT control and Gα_11_KO/Gα_q_-eKO mice received 15 mm incisional wound and closure was monitored over 18 days. A) Gross examination of the wounds in Gα_11_KO/Gα_q_-WT control (N = 15) and Gα_11_KO/Gα_q_-eKO (N = 10) mice. B) Initial wound size. Statistical significance determined by Student’s t-test. C) Kaplan-Meier survival curve of wound closure. Statistical significance was determined by log-rank test. D) Wound closure over time. Statistical significance was determined by two-way ANOVA. E) Wound half-life for Gα_11_KO/Gα_q_-WT control and Gα_11_KO/Gα_q_-eKO animals, as determined from the rate of wound closure over time. Statistical significance was determined by Student’s t-test, ***p<0.001.

The ability of keratinocytes to migrate into the wound bed is an essential step in cutaneous wound healing and essential for reepithelialization. As such, we reasoned that the pronounced migratory defect in Gα_11/q_-null animals might attenuate wound healing and lead to a delay in wound closure. Gα_11_KO/Gα_q_-WT control and Gα_11_KO/Gα_q_-eKO littermates were subjected to 15 mm dorsal cutaneous wounds and observed continuously during the wound healing process (Fig 4A). Initial wound sizes were nearly identical between Gα_11_KO/Gα_q_-WT control and Gα_11_KO/Gα_q_-eKO animals (Fig 4B). Despite this, Gα_11_KO/Gα_q_-eKO mice healed significantly later than their control littermates (Fig 4C). This delay in wound healing was directly attributed to loss of both epidermal Gα_q_ proteins, Gα_q_ and Gα_11_ as Gα_11_-null control animals alone healed similarly to wild-type animals (S2 Fig). Analysis of the rate of wound healing between Gα_11_KO/Gα_q_-WT control and Gα_11_KO/Gα_q_-eKO animals demonstrated a significant shift at all time points, leading to a highly significant difference in the wound healing curves (Fig 4D). Nonlinear regression of these curves revealed that the Gα_11_KO/Gα_q_-eKO animals had a wound half-life nearly twice that of Gα_11_KO/Gα_q_-WT control mice (Fig 4E). Together, this data indicates that the migratory defect incurred upon loss of Gα_q_ has significant impact on the rate of cutaneous wound healing and loss of epidermal Gα_q_ may lead to the development of chronic wounds.

### Gα_q_-mediated wound healing defects correlate with insensitivity to the regulation of differentiation signals

**Fig 5:**
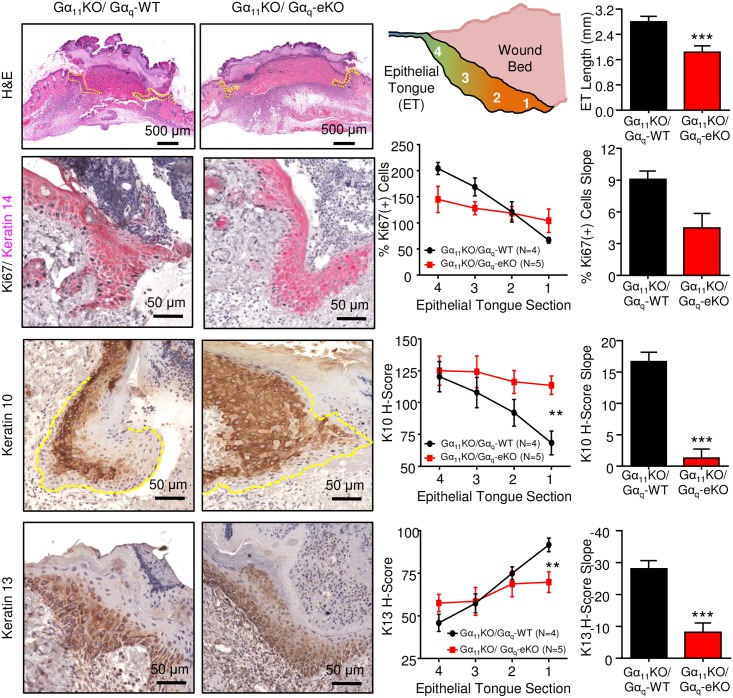
Loss of epidermal Gα_q_ correlates with defects in reepithelialization of cutaneous wounds. Top panel: the epithelial tongue as evidenced by H&E staining (yellow dashed line) is diagrammed and the length quantified. Representative stains and quantification of Ki67 (second panel), K10 (third panel), and K13 (bottom panel) are shown. All quantifications are based on an N = 4 mice per group and proliferation is shown relative to normal adjacent epithelium. Slope was determined by linear regression and statistical significance was determined by Student’s t-test, p<0.05, **p<0.01, ***p<0.001.

Defects in the keratinocyte migration impair subsequent reepithelialization phase of wound healing and can be seen by a failure to form an epithelial tongue adjacent to the wound bed. This was confirmed by H&E analysis, where the length of the migrating epithelial tongue in Gα_11_KO/Gα_q_-eKO cells was significantly smaller than their Gα_11_KO/Gα_q_-WT control counterparts (Fig 5, top row). While migrating cells closest to the wound bed do not demonstrate significant levels of proliferation, the trailing end of the epithelial tongue engages in robust proliferation to facilitate reepithelialization [28]. Compared to normal adjacent epithelium, WT cells demonstrate the spectrum of proliferation associated with this phase of wound healing, where the proximal segment is less proliferative than normal keratinocytes but a sharp doubling in proliferation can be seen at the supporting edge (Fig 5, second row). In contrast, KO cells demonstrate a relatively stable level of proliferation throughout the epithelial tongue that is not overtly different from the adjacent normal cells (Fig 5, second row). In the same manner, Gα_11_KO/Gα_q_-eKO animals demonstrate a profound inability to downregulate markers of differentiation at the leading edge of the epithelial tongue. While control littermates show a steady and significant decrease in the early differentiation marker K10 as the epithelial tongue approaches the wound bed, a nearly uniform expression of K10 is observed in all segments of the epithelial tongue of Gα_11_KO/Gα_q_-eKO animals (Fig 5, third row). This correlated to a nearly 13-fold reduction in slope for the K10 H-score across the epithelial tongue (Fig 5, third row). Defects in reepithelialization are associated with chronic wounds and malignant transformation in which reciprocal expression of K10 and K13 are often observed [29, 30]. Again, we observed that Gα_11_KO/Gα_q_-WT control animals had a steady, nearly linear increase in K13 H-score across the epithelial tongue that was abrogated in the Gα_11_KO/Gα_q_-eKO animals. While a slight increase was detected, it was still nearly 4 times less than that of the control animals. Together, this data indicates that the migratory defect that results from epidermal loss of Gα_q_ manifests in a failure of the epithelial tongue to migrate during wound healing. Further, it suggests that the deficiency in reepithelialization is due to an inability to engage the signaling pathways that originate within the wound bed.

## Discussion

Migration is a complex and highly orchestrated process that is essential for maintaining many cell functions under normal and pathologic conditions. Here, we demonstrate a novel role for Gα_q_ in facilitating acquisition of a stress-induced migratory phenotype in epidermal cells in the context of Gα_11_ loss. Under normal conditions, loss of Gα_11/q_ signaling does not demonstrate any overt effects but upon wounding, Gα_11/q_-null cells exhibit a remarkable inability to initiate the standard migratory response both *in vitro* and *in vivo*. This migratory defect correlates strongly with an inability to engage in the normal differentiation and proliferative programs that are initiated upon wounding and consequently lead to significantly delayed wound closure. These results have strong implications for our current understanding for the management of chronic wounds and aggressive malignancies whose pathologic hallmarks include the acquisition of a migratory phenotype.

Migration of keratinocytes, either collectively or as single cells, depends on the integration of internal and external cues to achieve directed movement [31]. These extracellular cues involve growth factors including members of the EGF, FGF, insulin, and VEGF families [32]. Gα proteins have been shown to be critical in the transactivation of many of these growth factor receptors and in some cases this transactivation has been shown to have pathologic consequences including tumor development and metastasis [33–35]. Despite the significant contribution of other Gα family members with migration, remarkably little is known regarding the specific role of Gα_q_ in epidermal motility. LPA has been shown to activate Gα_11/q_ signaling pathways and drive cell proliferation, differentiation, and migration in keratinocytes, similar to the effect observed upon TGF-β stimulation [36]. Conversely, transactivation of the insulin-like growth factor I receptor (IGF1R) by UTP-stimulated P2Y purinergic receptors inhibits PI3K activation in a Gα_q_-dependent manner, blocking subsequent keratinocyte migration [37, 38]. However, in other contexts, Gα_q_ is critical for activation of PI3K, MAPK, and other motility-associated pathways [39], suggesting that the role of Gα_q_ in migration may be to integrate multiple and sometimes conflicting extracellular signals to achieve appropriate cellular responses.

Wound healing is a clear example of a process that involves such an incorporation of multiple complex signaling networks. In order to restore barrier function, keratinocytes must coordinate migration, proliferation, and differentiation within a dynamic extracellular space. After acute injury, keratinocytes at the margin of wounds begin to migrate towards the wound bed. Proximity to the proinflammatory cytokines secreted at the wound bed leads to a number of phenotypical changes, including downregulation of proliferative and differentiation markers such as Ki67 and K10 [28]. At the same time, epidermal cells trailing the migrating keratinocytes engage in a hyperproliferative program to support the wound healing process [28]. Indeed, WT keratinocytes demonstrate a burst of proliferation at the trailing edge of the epithelial tongue that is nearly twice that of normal epithelium while simultaneously downregulating proliferation at the leading edge. Conversely, Gα_11/q_-null keratinocytes show a remarkably dampened response to stimuli, with an only slightly increased level of proliferation in the distal tongue section and a relatively unchanged level of proliferation at the leading edge. A similar trend is observed with respect to differentiation, as Gα_11/q_-null cells show little to no downregulation of K10, while WT cells actively engage in dedifferentiation at the wound bed.

Failure to enact the reepithelialization phase of wound healing due to diminished keratinocyte migration is clinically associated with chronic wounds. Cutaneous squamous cell carcinomas (SCCs) in particular have been shown to arise from precancerous lesions associated with chronic wounds and impaired wound healing paradigms [40]. Activation of the epithelial stem cell compartment is a critical element of wound healing, and epidermal loss of another Gα family member, Gα_s_, was associated with subsequent formation of basal cell carcinomas [41]. Therefore, chronic wounds demonstrating loss of Gα_q_ function may be more prone to development of precancerous and cancerous lesions in the skin.

In addition to activation of the stem cell compartment, chronic wounds and tumors share many other similar mechanistic and molecular manifestations, including irreversible loss of keratinocyte differentiation, uncontrolled proliferation, and enhanced migration [42]. Impaired signaling, proliferation, and migration of fibroblasts in chronic wounds have been observed and are in part attributable to decreased sensitivity to TGF-β1 [43]. Given the decreased responsiveness of Gα_11/q_-null cells to TGF-β1, this suggests that loss of Gα_q_-mediated signaling may contribute to the development of chronic wounds that could progress to SCCs. K13, a common marker of malignant progression, typically replaces expression of K10 during tumor progression and is also seen at higher levels at the leading edge of the epithelial tongue in wound healing [29, 30]. Only cells expressing Gα_q_ were able to differentially regulate expression of K13 (Fig 5), indicating that Gα_q_ is essential for transmitting the multilayered cues within the wound microenvironment. Taken together, it is tempting then to speculate then that under conditions of chronic inflammation and pathologic injury, loss of Gα_q_ may contribute to chronic wound formation and subsequent malignant transformation in the epithelial compartment.

Gα_q_ and Gα_11_ are integral members of the Gα family of proteins and play multifaceted roles in the regulation of gene expression, viability, and growth. Here, we propose that Gα_q_ proteins may have pleiotropic effects in the epithelial compartment under pathologic conditions to coordinate reepithelialization. Loss of epithelial Gα_11/q_ during the wound healing process manifests in a severe migratory phenotype punctuated by an inability of Gα_11/q_-null cells to differentially regulate proliferation and differentiation proximal to the wound bed. As such, Gα_q_ proteins are essential for the integration of the complex signaling networks in pathologic conditions and may provide a unique opportunity for clinical intervention.

## Supporting information

S1 Fig GPCR and Gaq-coupled GPCR ontologies are highly associated with keratinocyte migration. Expression levels of statistically significant differentially regulated genes involved in migration were downloaded and analyzed for ontologic similarities using the ENRICHR program. The GO Molecular Function ontology is represented according to the internally calculated p-value.
(TIF)

S2 Fig Wound healing in Gα_11_KO mice is not significantly different from wild-type mice. Wild-type C57B/L6 mice and Gα_11_KO/Gα_q_-WT mice were given 15 mm incisional wounds and closure was monitored over 18 days. A) Initial wound size. Statistical significance determined by Student’s t-test. B) Kaplan-Meier survival curve of wound closure. Statistical significance was determined by log-rank test. C) Wound closure over time. Statistical significance was determined by two-way ANOVA. D) Wound half-life for WT and Gα_11_KO as determined from the rate of wound closure over time. Statistical significance was determined by Student’s t-test, ns = not significant.
(TIF)

## References

[1] O'Hayre M, Degese MS, Gutkind JS. Novel insights into G protein and G protein-coupled receptor signaling in cancer. Curr Opin Cell Biol. 2014;27:126–35. Epub 2014/02/11. 10.1016/j.ceb.2014.01.005 24508914 PMC4021379

[2] Griewank K V S; Bastian BC. GNA11 (guanine nucleotide binding protein (G protein), alpha 11 (Gq class)). Atlas Genet Cytogenet Oncol Haematol. 2011;15(10):828–30.

[3] Kalinec G, Nazarali AJ, Hermouet S, Xu N, Gutkind JS. Mutated alpha subunit of the Gq protein induces malignant transformation in NIH 3T3 cells. Mol Cell Biol. 1992;12(10):4687–93. Epub 1992/10/01. 1328859 10.1128/mcb.12.10.4687-4693.1992 PMC360395

[4] O'Hayre M, Vazquez-Prado J, Kufareva I, Stawiski EW, Handel TM, Seshagiri S, et al The emerging mutational landscape of G proteins and G-protein-coupled receptors in cancer. Nat Rev Cancer. 2013;13(6):412–24. Epub 2013/05/04. 10.1038/nrc3521 23640210 PMC4068741

[5] Van Raamsdonk CD, Griewank KG, Crosby MB, Garrido MC, Vemula S, Wiesner T, et al Mutations in GNA11 in uveal melanoma. N Engl J Med. 2010;363(23):2191–9. 10.1056/NEJMoa1000584 21083380 PMC3107972

[6] Griewank KG, Schilling B, Scholz SL, Metz CH, Livingstone E, Sucker A, et al Oncogene status as a diagnostic tool in ocular and cutaneous melanoma. Eur J Cancer. 2016;57:112–7. 10.1016/j.ejca.2016.01.010 26918736

[7] Chattopadhyay C, Kim DW, Gombos DS, Oba J, Qin Y, Williams MD, et al Uveal melanoma: From diagnosis to treatment and the science in between. Cancer. 2016;122(15):2299–312. 10.1002/cncr.29727 26991400 PMC5567680

[8] Decatur CL, Ong E, Garg N, Anbunathan H, Bowcock AM, Field MG, et al Driver Mutations in Uveal Melanoma: Associations With Gene Expression Profile and Patient Outcomes. JAMA Ophthalmol. 2016;134(7):728–33. 10.1001/jamaophthalmol.2016.0903 27123562 PMC4966162

[9] Marinissen MJ, Gutkind JS. G-protein-coupled receptors and signaling networks: emerging paradigms. Trends Pharmacol Sci. 2001;22(7):368–76. Epub 2001/06/30. 11431032 10.1016/s0165-6147(00)01678-3

[10] Offermanns S, Zhao LP, Gohla A, Sarosi I, Simon MI, Wilkie TM. Embryonic cardiomyocyte hypoplasia and craniofacial defects in G alpha q/G alpha 11-mutant mice. EMBO J. 1998;17(15):4304–12. 10.1093/emboj/17.15.4304 9687499 PMC1170764

[11] Offermanns S, Hashimoto K, Watanabe M, Sun W, Kurihara H, Thompson RF, et al Impaired motor coordination and persistent multiple climbing fiber innervation of cerebellar Purkinje cells in mice lacking Galphaq. Proc Natl Acad Sci U S A. 1997;94(25):14089–94. 9391157 10.1073/pnas.94.25.14089 PMC28437

[12] Wettschureck N, Rutten H, Zywietz A, Gehring D, Wilkie TM, Chen J, et al Absence of pressure overload induced myocardial hypertrophy after conditional inactivation of Galphaq/Galpha11 in cardiomyocytes. Nat Med. 2001;7(11):1236–40. 10.1038/nm1101-1236 11689889

[13] Mikelis CM, Simaan M, Ando K, Fukuhara S, Sakurai A, Amornphimoltham P, et al RhoA and ROCK mediate histamine-induced vascular leakage and anaphylactic shock. Nat Commun. 2015;6:6725 Epub 2015/04/11. 10.1038/ncomms7725 25857352 PMC4394241

[14] Van Raamsdonk CD, Fitch KR, Fuchs H, de Angelis MH, Barsh GS. Effects of G-protein mutations on skin color. Nat Genet. 2004;36(9):961–8. 10.1038/ng1412 15322542 PMC7341985

[15] Fitch KR, McGowan KA, van Raamsdonk CD, Fuchs H, Lee D, Puech A, et al Genetics of dark skin in mice. Genes Dev. 2003;17(2):214–28. 10.1101/gad.1023703 12533510 PMC195979

[16] Korhonen H, Fisslthaler B, Moers A, Wirth A, Habermehl D, Wieland T, et al Anaphylactic shock depends on endothelial Gq/G11. J Exp Med. 2009;206(2):411–20. Epub 2009/01/28. 10.1084/jem.20082150 19171764 PMC2646572

[17] Andl T, Ahn K, Kairo A, Chu EY, Wine-Lee L, Reddy ST, et al Epithelial Bmpr1a regulates differentiation and proliferation in postnatal hair follicles and is essential for tooth development. Development. 2004;131(10):2257–68. Epub 2004/04/23. 10.1242/dev.01125 15102710

[18] Schrage R, Schmitz AL, Gaffal E, Annala S, Kehraus S, Wenzel D, et al The experimental power of FR900359 to study Gq-regulated biological processes. Nat Commun. 2015;6:10156 10.1038/ncomms10156 26658454 PMC4682109

[19] Dlugosz AA, Glick AB, Tennenbaum T, Weinberg WC, Yuspa SH. Isolation and utilization of epidermal keratinocytes for oncogene research. Methods Enzymol. 1995;254:3–20. Epub 1995/01/01. 8531694 10.1016/0076-6879(95)54003-2

[20] Romer J, Bugge TH, Pyke C, Lund LR, Flick MJ, Degen JL, et al Impaired wound healing in mice with a disrupted plasminogen gene. Nat Med. 1996;2(3):287–92. Epub 1996/03/01. 8612226 10.1038/nm0396-287

[21] Feng X, Degese MS, Iglesias-Bartolome R, Vaque JP, Molinolo AA, Rodrigues M, et al Hippo-independent activation of YAP by the GNAQ uveal melanoma oncogene through a trio-regulated rho GTPase signaling circuitry. Cancer Cell. 2014;25(6):831–45. Epub 2014/06/03. 10.1016/j.ccr.2014.04.016 24882515 PMC4074519

[22] Vaque JP, Dorsam RT, Feng X, Iglesias-Bartolome R, Forsthoefel DJ, Chen Q, et al A genome-wide RNAi screen reveals a Trio-regulated Rho GTPase circuitry transducing mitogenic signals initiated by G protein-coupled receptors. Mol Cell. 2013;49(1):94–108. Epub 2012/11/28. 10.1016/j.molcel.2012.10.018 23177739 PMC3545055

[23] Offermanns S, Toombs CF, Hu YH, Simon MI. Defective platelet activation in G alpha(q)-deficient mice. Nature. 1997;389(6647):183–6. 10.1038/38284 9296496

[24] Cheng CF, Fan J, Bandyopahdhay B, Mock D, Guan S, Chen M, et al Profiling motility signal-specific genes in primary human keratinocytes. J Invest Dermatol. 2008;128(8):1981–90. Epub 2008/03/08. 10.1038/jid.2008.34 18323786 PMC6554730

[25] Hata A, Chen YG. TGF-beta Signaling from Receptors to Smads. Cold Spring Harb Perspect Biol. 2016. 10.1101/cshperspect.a022061 PMC5008074 27449815

[26] Koivisto L, Heino J, Hakkinen L, Larjava H. Integrins in Wound Healing. Adv Wound Care (New Rochelle). 2014;3(12):762–83. 25493210 10.1089/wound.2013.0436 PMC4250945

[27] Hameedaldeen A, Liu J, Batres A, Graves GS, Graves DT. FOXO1, TGF-beta regulation and wound healing. Int J Mol Sci. 2014;15(9):16257–69. 10.3390/ijms150916257 25226535 PMC4200873

[28] Usui ML, Mansbridge JN, Carter WG, Fujita M, Olerud JE. Keratinocyte migration, proliferation, and differentiation in chronic ulcers from patients with diabetes and normal wounds. J Histochem Cytochem. 2008;56(7):687–96. Epub 2008/04/17. 10.1369/jhc.2008.951194 18413645 PMC2430161

[29] Betz P, Nerlich A, Tubel J, Penning R, Eisenmenger W. The time-dependent expression of keratins 5 and 13 during the reepithelialization of human skin wounds. Int J Legal Med. 1993;105(4):229–32. Epub 1993/01/01. 7679282 10.1007/BF01642799

[30] Glick AB, Kulkarni AB, Tennenbaum T, Hennings H, Flanders KC, O'Reilly M, et al Loss of expression of transforming growth factor beta in skin and skin tumors is associated with hyperproliferation and a high risk for malignant conversion. Proc Natl Acad Sci U S A. 1993;90(13):6076–80. Epub 1993/07/01. 7687059 10.1073/pnas.90.13.6076 PMC46870

[31] Friedl P, Wolf K. Plasticity of cell migration: a multiscale tuning model. J Cell Biol. 2010;188(1):11–9. Epub 2009/12/03. 10.1083/jcb.200909003 19951899 PMC2812848

[32] Seeger MA, Paller AS. The Roles of Growth Factors in Keratinocyte Migration. Adv Wound Care (New Rochelle). 2015;4(4):213–24. Epub 2015/05/07. 25945284 10.1089/wound.2014.0540 PMC4397993

[33] Filardo EJ, Quinn JA, Bland KI, Frackelton AR Jr. Estrogen-induced activation of Erk-1 and Erk-2 requires the G protein-coupled receptor homolog, GPR30, and occurs via trans-activation of the epidermal growth factor receptor through release of HB-EGF. Mol Endocrinol. 2000;14(10):1649–60. Epub 2000/10/24. 10.1210/mend.14.10.0532 11043579

[34] Hart S, Fischer OM, Prenzel N, Zwick-Wallasch E, Schneider M, Hennighausen L, et al GPCR-induced migration of breast carcinoma cells depends on both EGFR signal transactivation and EGFR-independent pathways. Biol Chem. 2005;386(9):845–55. Epub 2005/09/17. 10.1515/BC.2005.099 16164409

[35] Prenzel N, Zwick E, Daub H, Leserer M, Abraham R, Wallasch C, et al EGF receptor transactivation by G-protein-coupled receptors requires metalloproteinase cleavage of proHB-EGF. Nature. 1999;402(6764):884–8. Epub 2000/01/06. 10.1038/47260 10622253

[36] Sauer B, Vogler R, Zimmermann K, Fujii M, Anzano MB, Schafer-Korting M, et al Lysophosphatidic acid interacts with transforming growth factor-beta signaling to mediate keratinocyte growth arrest and chemotaxis. J Invest Dermatol. 2004;123(5):840–9. Epub 2004/10/16. 10.1111/j.0022-202X.2004.23458.x 15482469

[37] Taboubi S, Milanini J, Delamarre E, Parat F, Garrouste F, Pommier G, et al G alpha(q/11)-coupled P2Y2 nucleotide receptor inhibits human keratinocyte spreading and migration. Faseb J. 2007;21(14):4047–58. Epub 2007/07/05. 10.1096/fj.06-7476com 17609252

[38] Taboubi S, Garrouste F, Parat F, Pommier G, Faure E, Monferran S, et al Gq-coupled purinergic receptors inhibit insulin-like growth factor-I/phosphoinositide 3-kinase pathway-dependent keratinocyte migration. Mol Biol Cell. 2010;21(6):946–55. Epub 2010/01/22. 10.1091/mbc.E09-06-0497 20089844 PMC2836975

[39] Dorsam RT, Gutkind JS. G-protein-coupled receptors and cancer. Nat Rev Cancer. 2007;7(2):79–94. Epub 2007/01/26. 10.1038/nrc2069 17251915

[40] Leiter U, Eigentler T, Garbe C. Epidemiology of skin cancer. Adv Exp Med Biol. 2014;810:120–40. 25207363 10.1007/978-1-4939-0437-2_7

[41] Iglesias-Bartolome R, Torres D, Marone R, Feng X, Martin D, Simaan M, et al Inactivation of a Galphas-PKA tumour suppressor pathway in skin stem cells initiates basal-cell carcinogenesis. Nat Cell Biol. 2015;17(6):793–803. Epub 2015/05/12. 10.1038/ncb3164 25961504 PMC4449815

[42] Dvorak HF. Tumors: wounds that do not heal. Similarities between tumor stroma generation and wound healing. N Engl J Med. 1986;315(26):1650–9. Epub 1986/12/25. 10.1056/NEJM198612253152606 3537791

[43] Kim BC, Kim HT, Park SH, Cha JS, Yufit T, Kim SJ, et al Fibroblasts from chronic wounds show altered TGF-beta-signaling and decreased TGF-beta Type II receptor expression. J Cell Physiol. 2003;195(3):331–6. 10.1002/jcp.10301 12704642

