# Supplementary figures and images for: Epidermal loss of Gαq confers a migratory and differentiation defect in keratinocytes
Source: PLoS One. 2017 Mar 16;12(3):e0173692. doi: 10.1371/journal.pone.0173692 (PMC5354386; doi:10.1371/journal.pone.0173692)

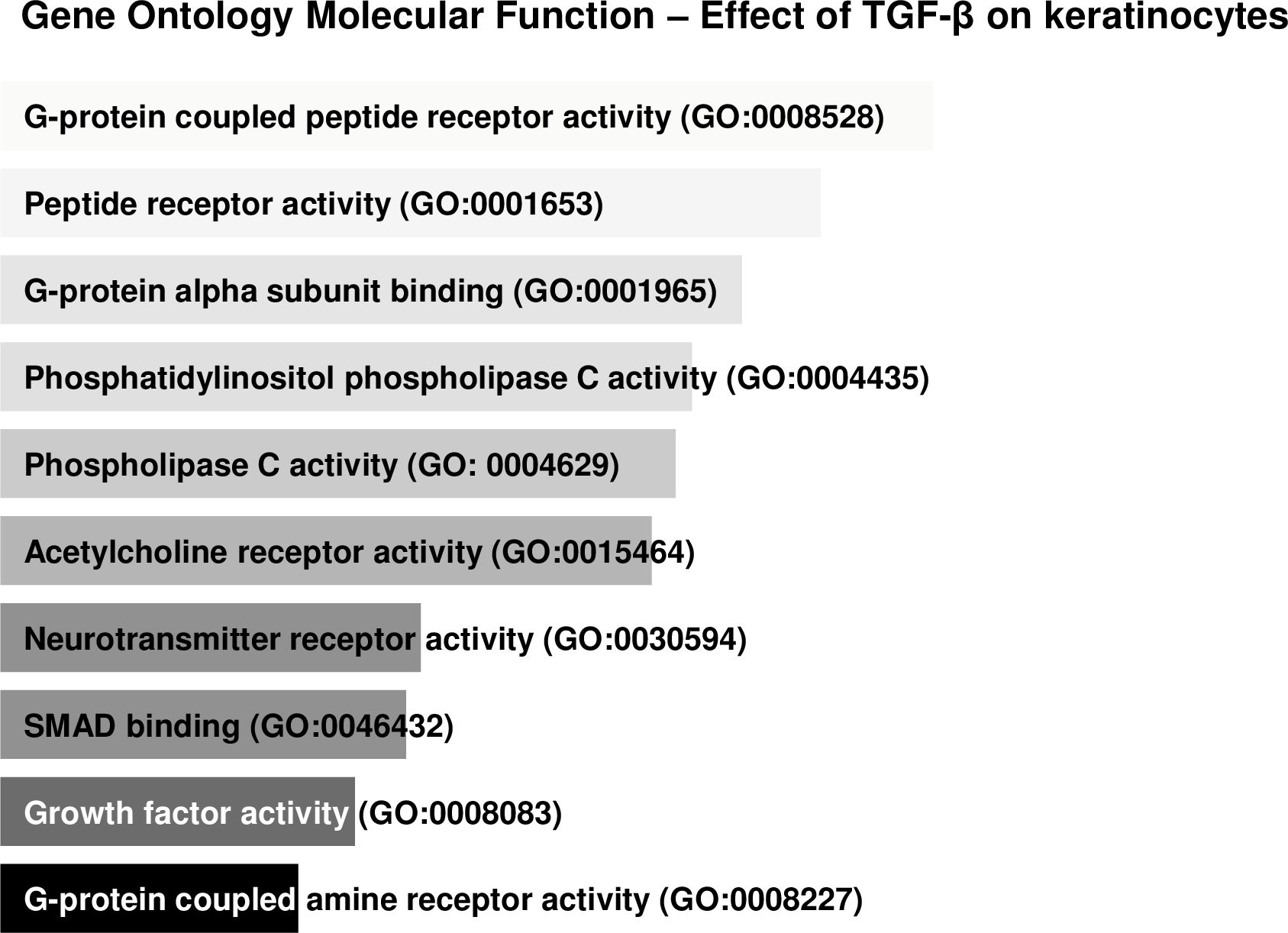

Supplement: S1 Fig — Expression levels of statistically significant differentially regulated genes involved in migration were downloaded and analyzed for ontologic similarities using the ENRICHR program. The GO Molecular Function ontology is represented according to the internally calculated p-value. (TIF) [file pone.0173692.s001.tif]

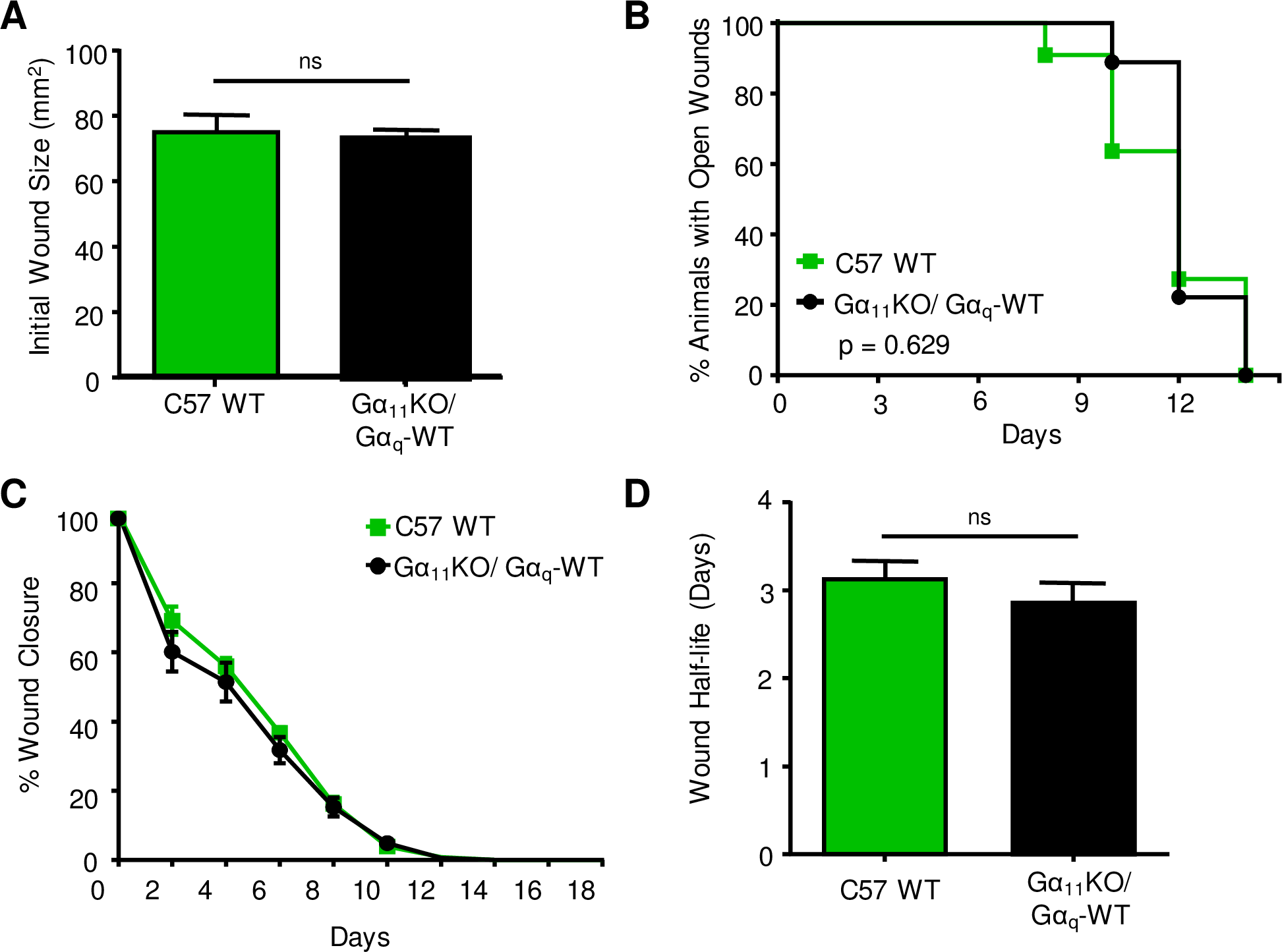

Supplement: S2 Fig — Wild-type C57B/L6 mice and Gα11KO/Gαq-WT mice were given 15 mm incisional wounds and closure was monitored over 18 days. A) Initial wound size. Statistical significance determined by Student’s t-test. B) Kaplan-Meier survival curve of wound closure. Statistical significance was determined by log-rank test. C) Wound closure over time. Statistical significance was determined by two-way ANOVA. D) Wound half-life for WT and Gα11KO as determined from the rate of wound closure over time. Statistical significance was determined by Student’s t-test, ns = not significant. (TIF) [file pone.0173692.s002.tif]
